# Identification of two novel null variants in *CLN8* by targeted next-generation sequencing: first report of a Chinese patient with neuronal ceroid lipofuscinosis due to *CLN8* variants

**DOI:** 10.1186/s12881-018-0535-7

**Published:** 2018-02-08

**Authors:** Zhijie Gao, Hua Xie, Qian Jiang, Nan Wu, Xiaoli Chen, Qian Chen

**Affiliations:** 1 Department of Neurology, Affiliated Children’s Hospital of Capital Institute of Pediatrics, No. 2, Yabao Road, Chaoyang District, Beijing, 100020 China; 2 Department of Medical Genetics, Beijing Municipal Key Laboratory of Child Development and Nutriomics, Capital Institute of Pediatrics, Beijing, 100020 China; 3 Beijing Key Laboratory for Genetic Research of Skeletal Deformity, Beijing, 100020 China

**Keywords:** Neuronal ceroid lipofuscinoses, *CLN8*, Novel null variant

## Abstract

**Background:**

Neuronal ceroid lipofuscinoses (NCLs) are one of the most frequent childhood-onset neurodegenerative pathologies characterized by seizures, progressive cognitive decline, motor impairment and loss of vision. For the past two decades, more than 430 variants in 13 candidate genes have been identified in the affected patients. Most of the variants were almost exclusively reported in Western patients, and very little clinical and genetic information was available for Chinese patients.

**Case presentation:**

We report a Chinese boy whose clinical phenotypes were suspected to be NCL, including intractable epilepsy, cognitive and motor decline and progressive vision loss. Using targeted next-generation sequencing, two novel null variants in *CLN8* (c.298C > T, p.Gln100Ter; c.551G > A, p.Trp184Ter) were detected in this patient in *trans* model. These two variants were interpreted as pathogenic according to the variant guidelines of the American College of Medical Genetics and Genomics.

**Conclusions:**

This is the first case report of NCL due to *CLN8* variants in China. Our findings expand the variant diversity of *CLN8* and demonstrate the tremendous diagnosis value of targeted next-generation sequencing for pediatric NCLs.

**Electronic supplementary material:**

The online version of this article (10.1186/s12881-018-0535-7) contains supplementary material, which is available to authorized users.

## Background

The neuronal ceroid lipofuscinoses (NCLs) are a group of inherited neurodegenerative disorders characterized by epilepsy, progressive cognitive and motor decline, loss of vision, dementia, and usually reduced life expectancy [1]. NCL has been recognized as one of the most frequent childhood-onset neurodegenerative pathologies, with a prevalence of 1:1,000,000 to 1:14,000 worldwide [2]. NCLs are classified into six subtypes according to the primary onset of symptoms, and broad phenotypic variance has been reported in different subtypes [3]. The accumulation of autofluorescent lysosomal storage material in the central nervous system is a key pathological finding of NCLs. Several possible candidate genes are involved in this process, including genes encoding lysosomal enzymes (*CLN1/PPT1, CLN2/TPP1, CLN10/CTSD, CLN13/CTSF*), genes encoding a soluble lysosomal protein (*CLN5*) and genes encoding a protein in the secretory pathway (*CLN11/GRN*). In addition, genes encoding cytoplasmic proteins (*CLN4/DNAJC5*, *CLN14/KCTD7*) and transmembrane proteins (*CLN3*, *CLN6*, *CLN7/MFSD8*, *CLN8*, *CLN12/ATP13A2*) are also associated with NCLs [4]. In addition to phenotypic and genetic heterogeneity, allelic heterogeneity of the same gene has also been previously described in affected patients [5], indicating that precise diagnosis of NCLs may mainly rely on molecular genetic testing.

To date, more than 430 pathogenic variants in the above 13 candidate genes have been reported in human NCLs, and most have been registered in the NCL Mutation Database (http://www.ucl.ac.uk/ncl/) [6]. Seventy-four patients were attributed to pathogenic variants in the *CLN8* gene (OMIM #607837). Sporadic NCL patients have been occasionally reported in Chinese [7–9], however, the variant spectrum in Chinese NCL patients is still unknown. In addition, variants in *CLN8* have not been identified in Chinese NCL patients. Here we reported our diagnostic experience of a Chinese boy presenting typical clinical manifestation of NCL. We identified two novel pathogenic variants in *CLN8* using targeted next-generation sequencing and completed genetic diagnosis for this patient in a very short turn-around time. To the best of our knowledge, this is the first reported NCL patient due to *CLN8* variants in China. Our report demonstrates the absolute diagnosis advantage of high-throughput genomic sequencing for pediatric neurodegenerative disease with high phenotypic and genetic heterogeneity, making patients move to the precise genotyping.

## Case presentation

### Clinical information

The study was approved by the ethics committee of Capital Institute of Pediatrics. Written informed consent was obtained from the patient’s parents for the publication of this report and any accompanying images. The patient was a Chinese boy aged 8 years and 8 months. He was born in a non-consanguineous family, with normal pregnancy and perinatal history. No family history of epilepsy was recorded. He had one healthy older sister.

This boy developed seizures at the age of 4 years, which was relieved after treatment with Levetiracetam. He initially had normal social and language development, but showed motor regression after seizure attacks, such as unsteady gait and tiptoeing. At the age of seven, a progressive regression in cognitive skills, language and motor abilities was noticed by the parents. The boy began producing unintelligible words and finally developed to autism-like symptoms (no language communication with family members). Since then, his seizures cannot be controlled by multi-antiepileptic drugs, including Levetiracetam, sodium valproate, Oxcarbazepine and Clonazepam.

**Fig. 1:**
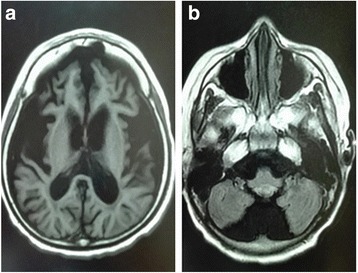
T1-weighted MRI images for the patient showed cerebral (**a**) and cerebellar (**b**) atrophy

The patient experienced progressive visual loss since the age of seven. He was completely bed-bound with very poor vision and uncontrolled seizure at the age of eight and a half. Gradually, he became unable to perform activities without assistance. Due to the progressive dysphagia, a semi-liquid diet was arranged for him. Brain MRI showed diffuse cerebral and cerebellar atrophy (Fig. 1a, b), and the electroencephalogram revealed irregular and slow background activity and a high incidence of generalized sequences of atypical spike-wave discharges. Based on the neurological symptoms and brain radiological features, this patient was clinically suspected to be NCL.

### Targeted panel sequencing and data analysis

Genomic DNA was extracted from peripheral blood of the patient and his parents (DNA Blood Mini Kit, Qiagen, Hilden, Germany). Targeted panel sequencing was performed for the 13 known candidate genes responsible for NCLs (*CLN1, CLN2, CLN3, CLN4, CLN5, CLN6, CLN7, CLN8, CLN10, CLN11, CLN12, CLN13* and *CLN14*). Because the patient presented uncontrolled seizure, the epilepsy panel (462 relevant genes in Additional file 1) was also performed to exclude other causative variants. Both exon and exon-intron boundary regions (10 bp) of target genes were enriched by a commercial enrichment kit (SureSelect, Agilent Technologies Inc., Palo Alto, CA) and sequenced on the Illumina GAIIx platform. Raw data was first mapped to the reference human genome version hg19 (200,902 release, http://genome.ucsc.edu/), and then analyzed by the DNASTAR software (Madison, WI, USA) and visualized by the NextGENe software version 2.1.1.1 (SoftGenetics, State College, PA).

Single nucleotide variants (SNVs) with minor allele frequency (> 1%) in the dbSNP database (http://www.ncbi.nlm.nih.gov/snp) or the 1000 genome dataset (http://browser.1000genomes.org/index.html), the NHLBI Exome Sequencing Project (http://evs.gs.washington.edu/EVS/) were not recognized as rare SNVs. The ExAC database was used to confirm the novelty of rare SNVs.

### Results

**Fig. 2:**
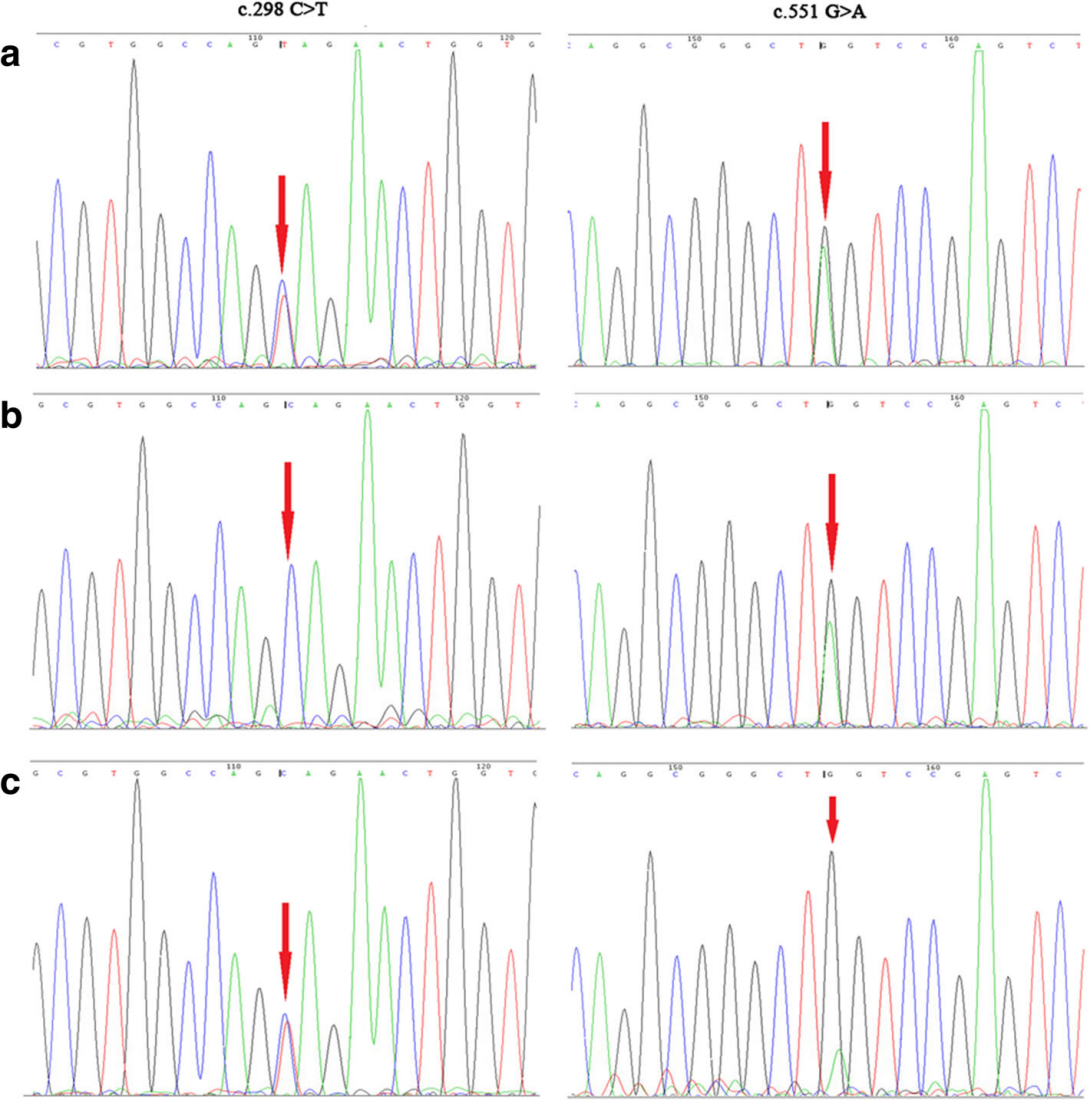
Sanger traces for PCR products of the patient and his parents. **a** Sanger traces for PCR products of the patient showed the two heterozygous null variants in *CLN8* (c.298C > T, p.Gln100Ter; c.551G > A, p.Trp184Ter). **b** Sanger traces for PCR products of his father, which confirmed that the c.298 C > T variant was inherited from the father. **c** Sanger traces for PCR products of his mother, which confirmed that the c.551 G > A variant was inherited from the mother

We detected a total of 523 rare variants from two panel sequencing. We excluded 507 Chinese-specific but benign variants by filtering against the sequencing data from 200 non-NCL patients and 50 normal children. Because the purpose of the current study was to reach clinical diagnosis, we strictly followed the guidelines of the American College of Medical Genetics and Genomics (ACMG) [10] and preferentially considered null variants (stop-gain, frameshift, canonical splicing variant and exonic deletion). Among the remaining 16 rare SNVs, there were no other genes carrying homozygous or compound heterozygous variants, except for two variants in *CLN8*. The two variants (chr8:1,719,518, C > T and chr8:1,728,423, G > A) of *CLN8* (NM_018941.3) are stop-gain substitutions resulting in truncated transcripts in exon 2 and exon 3 (c.298C > T, p.Gln100Ter and c.551G > A, p.Trp184Ter, respectively) and were annotated as null variants. Sanger sequencing validated the reality of inheritance of the two variants (Fig. 2). Each variant was inherited from one of the parents (in *trans* model, Fig. 2), which was coincident with the recessive inheritance model of NCLs. These two null variants were interpreted as “pathogenic” according to the variant guidelines of ACMG based upon the following criteria: predicted as null variants (PVS1), absent in population databases (PM1), and in *trans* for recessive disorders (PM3). Therefore, we summarized that the compound null variants in *CLN8* likely contribute to the NCL phenotype in this patient.

## Discussion and conclusions

NCLs are a group of heterogeneous disorders characterized clinically by visual failure, behavioral problems, onset of seizure, cognitive regression and motor impairment. For the clinician, the key suggestive sign for NCLs is electron microscopy examination showing ultra-structural lipofuscinic pigments [11]. Reduced enzyme activity is another suggestive sign for some subtypes of NCLs [12]. Despite these clinical clues, NCLs remain a challenge for neurologists, especially for the pediatric neurologist, because the clinical signs in young children or toddlers are subtle and often overlap with other congenital neurodegenerative diseases, such as mitochondrial disorders or early-onset Parkinsonism [11]. For the geneticist, the variants in different candidate genes could cause the same NCL subtype. Meanwhile, variants in the same candidate gene (but in different alleles) could cause different NCL subtypes [5]. Thus, screening all relevant disease-related genes is an efficient and simple procedure to achieve precise diagnosis.

In this study, we reported a Chinese patient with NCL caused by two novel null variants in *CLN8*. Both variants are interpreted as “pathogenic” according to the guidelines of ACMG. These two variants are located close to the transmembrane residues and predicted to cause invalid proteins due to the truncated transmembrane domain. To our knowledge, this is the first reported Chinese NCL patient with *CLN8* variants and the second reported Asian NCL patient with *CLN8* variants [13]. In general, the variants in *CLN8* are associated with two different phenotypes: (1) the EPMR phenotype (MIM#610003, epilepsy with progressive mental retardation, also called as Northern Epilepsy), which is characterized by normal early development, onset of drug resistance and generalized tonic-clonic seizure between the ages of 5 and 10 years; visual loss is not a prominent feature [14, 15]; and (2) late-infantile NCL (LI-NCL) phenotype, in which the onset of seizure is earlier and the disease progression is more rapid than EPMR [16–18]. This Chinese patient had suffered from intractable epilepsy after 4 years old, and his cognitive function and vision have progressively deteriorated after the epilepsy attack. Combining the phenotype and genetic testing results, the patient in our study was precisely diagnosed as variant LI-NCL (vLI-NCL).

So far, 74 NCL patients have been attributed to the pathogenic variants in *CLN8*, and most patients are from Finland and Turkey. The most frequent variants in *CLN8* were missense variants. The variants carried by the patient of our study were two novel null mutations (stop-gain), which have never been reported. The correlation between the phenotypic severity and variant category of *CLN8* remains unclear. However, the discoveries from patients with *CLN6* variants have provided some clues. In general, the null variant of *CLN6* results in severe clinical symptoms that occurs in the late infantile years, presenting with seizure, followed by severe vision loss, ataxia, mental regression and early death [5, 19]. In contrast, the missense variant results in a milder and early-adult-onset form characterized by progressive myoclonic epilepsy alone without visual failure [20]. We reviewed the NCL Mutation Database (http://www.ucl.ac.uk/ncl/) and the updated literature to analyze the phenotype-variant correlation for the NCL patients with *CLN8* variants [17, 21–24]. We found that the patients carrying *CLN8* null variants presented earlier onset and more progressive disease course than the patients carrying *CLN8* missense variants. We also reviewed the clinical phenotype of patients carrying *CLN8* bi-allelic or uni-allelic null variants. As Table 1 shows, seven NCL patients carrying null *CLN8* variants were retrieved and three of them carried bi-allelic null variants. All are Western patients and presented with LI-NCL or vLI-NCL, not EPMR. Our Chinese patient also suffered from intractable epilepsy, progressive vision loss, cognitive impairment and dysphagia, which is similar to that of Western patients. In addition, we found that variants in different domains of CLN8 protein cause same phenotypic severity of NCLs, suggesting that the *CLN8* variant does not have allelic heterogeneity for the NCL phenotype. We presumed that modifier variants in other NCL candidate genes or variants in the whole genome may be involved in the phenotypic variability of NCLs.

**Table 1:** The NCL phenotypes/subtypes in patients carrying *CLN8* null variants

DB Patient ID^a^	Allele 1 nt (aa)	Allele 2 nt (aa)	Phenotype	Age at onset	Histology	Origin/Residence	Reference
Pa-*CLN8*.033	c.88delG (p.Ala30fsX20)	c.88delG (p.Ala30fsX20)	vLI-NCL	3y	CL/FP	Turkey	Ranta et al., 2004 [21]
Pa-*CLN8*.035	c.66delG (p.Gly22SerfsX5)	c.581A > G (p.Gln194Arg)	vLI-NCL	4y	CL/FP	Italy	Cannelli et al., 2006 [22]
Pa-*CLN8*.036	c.66delG (p.Gly22SerfsX5)	c.473A > G (p.Tyr158Cys)	vLI-NCL	3.5y	CL/FP	Italy	Cannelli et al., 2006 [22]
Pa-*CLN8*.043	c.544-2566_590del2613 (p.Ala182AspfsX49)	c.544-2566_590del2613 (p.Ala182AspfsX49)	vLI-NCL	2.5y	CL/FP	Turkey	Reinhardt et al., 2010 [17]
Pa-CLN8.068	c.562_563delCT (p.Leu188ValfsX58)	8p23.3 terminal deletion	vLI-NCL	4y	FP (lymphocytes), CL, RL (skin)	Ireland	Allen et al. 2012 [24]
Pa-*CLN8*.073	c.763C > T	8p23.3 deletion, 235 Kb	vLI-NCL	NA	NA	UK	R. Williams pers. comm
Pa-*CLN8*.074	c.728 T > C	8p23.3 deletion, 54 Kb	vLI-NCL	NA	NA	UK	R. Williams pers. comm
This study	c.298 C > T (p.Gln100Ter)	c.551 G > A(p.Trp184Ter)	vLI-NCL	4y	NA	Chinese	

NCL patients carrying *CLN8* bi-allelic or uni-allelic null variant was included. *CLN8*: NM_018941.3. Null variant includes frameshift, splicing or non-sense mutation

*LI* late infantile (2–4 y), *vLI* varaint late infantile (3–7.5 y), *NA* not available, *CL* curvilinear, *RL* rectilinear, *FP* fingerprint

^a^: the ID in the NCL Mutation Database (http://www.ucl.ac.uk/ncl/)

In the past, candidate genes were tested one by one in the clinical diagnosis laboratory. However, this approach is time and labor consuming. With the broad development of next-generation sequencing techniques, target panel sequencing and whole exome/genome sequencing have been accepted as efficient and affordable approaches for genetic diagnosis in Mendelian recessive diseases and are particularly suitable for pediatric neurodegenerative diseases with high phenotypic and genetic heterogeneity, such as NCLs [25]. Previous studies have also demonstrated that target panel sequencing and whole exome sequencing are appropriate methods for undiagnosed pediatric neurodevelopmental disorders [26–28]. The primary results from the Epi4K/EuroEPINOMICS study revealed that trio-based next-generation sequencing provided a clear genetic etiologic diagnosis for approximately 12% of 356 patients with epilepsy [26]. In addition, whole exome sequencing can identify causative variants in 24–33% cases with unknown intellectual disability [27, 28]. In this study, the NCL candidate gene panel sequencing was chosen for the clinical suspicious NCL patient. The molecular diagnosis was reached within a very short turn-round time (3 weeks), suggesting that targeted sequencing is a powerful and efficient approach for clinical genetic laboratories to rapidly determine the molecular basis of NCLs and other analogous developmental disorders.

This study described a Chinese NCL patient who was diagnosed by targeted next-generation sequencing. This is the first reported Chinese patient presenting vLI-NCL phenotypes caused by two novel null variants in *CLN8*. Our findings expanded the variant diversity of *CLN8* and proved the utility value of targeted next-generation sequencing for pediatric NCLs.

## Additional file

Additional file 1: The gene list of epilepsy panel. (XLSX 45 kb)

## Abbreviations

ACMG: American College of Medical Genetics and Genomics
LI-NCL: late-infantile NCL
NCLs: Neuronal ceroid lipofuscinoses
vLI-NCL: variant LI-NCL
